# Prior Stroke and Age Predict Acute Ischemic Stroke Among Hospitalized COVID-19 Patients: A Derivation and Validation Study

**DOI:** 10.3389/fneur.2021.741044

**Published:** 2021-10-04

**Authors:** Teng J. Peng, Adam S. Jasne, Michael Simonov, Safa Abdelhakim, Gbambele Kone, Yee Kuang Cheng, Melissa Rethana, Karan Tarasaria, Alison L. Herman, Anna D. Baker, Shadi Yaghi, Jennifer A. Frontera, Lauren H. Sansing, Guido J. Falcone, Serena Spudich, Joseph Schindler, Kevin N. Sheth, Richa Sharma

**Affiliations:** ^1^Department of Neurology, Yale University School of Medicine, New Haven, CT, United States; ^2^Department of Internal Medicine, Yale University School of Medicine, New Haven, CT, United States; ^3^Department of Neurology, New York University Langone Health, New York, NY, United States; ^4^Department of Neurology, Brown University School of Medicine, Providence, RI, United States

**Keywords:** ischemic stroke, COVID-19, stroke of young, validation study, platelets, prior stroke

## Abstract

**Objectives:** Our objective was to identify characteristics associated with having an acute ischemic stroke (AIS) among hospitalized COVID-19 patients and the subset of these patients with a neurologic symptom.

**Materials and Methods:** Our derivation cohort consisted of COVID-19 patients admitted to Yale-New Haven Health between January 3, 2020 and August 28, 2020 with and without AIS. We also studied a sub-cohort of hospitalized COVID-19 patients demonstrating a neurologic symptom with and without an AIS. Demographic, clinical, and laboratory results were compared between AIS and non-AIS patients in the full COVID-19 cohort and in the sub-cohort of COVID-19 patients with a neurologic symptom. Multivariable logistic regression models were built to predict ischemic stroke risk in these two COVID-19 cohorts. These 2 models were externally validated in COVID-19 patients hospitalized at a major health system in New York. We then compared the distribution of the resulting predictors in a non-COVID ischemic stroke control cohort.

**Results:** A total of 1,827 patients were included in the derivation cohort (AIS *N* = 44; no AIS *N* = 1,783). Among all hospitalized COVID-19 patients, history of prior stroke and platelet count ≥ 200 × 1,000/μL at hospital presentation were independent predictors of AIS (derivation AUC 0.89, validation AUC 0.82), irrespective of COVID-19 severity. Among hospitalized COVID-19 patients with a neurologic symptom (*N* = 827), the risk of AIS was significantly higher among patients with a history of prior stroke and age <60 (derivation AUC 0.83, validation AUC 0.81). Notably, in a non-COVID ischemic stroke control cohort (*N* = 168), AIS patients were significantly older and less likely to have had a prior stroke, demonstrating the uniqueness of AIS patients with COVID-19.

**Conclusions:** Hospitalized COVID-19 patients who demonstrate a neurologic symptom and have either a history of prior stroke or are of younger age are at higher risk of ischemic stroke.

## Introduction

As of March 2021, the global pandemic caused by coronavirus disease 2019 (COVID-19) has infected more than 120 million individuals worldwide and has resulted in more than 2.6 million deaths (1). Although COVID-19 commonly manifests as a respiratory illness, it is also linked with an increased risk of cerebrovascular disease by multiple potential pathophysiologic mechanisms, including inflammation, hypercoagulability, and endotheliopathy (2–5). Among patients with COVID-19 in a cohort in Wuhan, China, those with a history of ischemic stroke had more co-morbidities, lower platelet and leukocyte counts, and higher D-dimer, pro-brain natriuretic peptide, and interleukin-6 levels compared to their counterparts without a prior history of stroke (6). A study performed at the New York City system hospitals also noted that COVID-19 patients with strokes vs. non-COVID-19 patients with strokes were more likely to be younger, male, and white (7). Data regarding predictors of ischemic stroke among all COVID patients and those with neurologic symptoms are very limited. Strokes in the context of COVID-19 have been previously associated with increased mortality and morbidity (8–10). Given these observations, there is a need to better predict, diagnose, and prevent ischemic strokes in patients with COVID-19.

Multiple guidelines regarding management of stroke in patients with COVID-19 have been released, but there is currently no means by which to stratify the risk of ischemic stroke among patients infected with the SARS-CoV-2 virus (11–13). Specific clinical and serologic biomarkers to predict the risk of stroke would facilitate implementation of preventative and diagnostic measures to optimize outcomes for patients with COVID-19. In this analysis, we aimed to identify risk factors of acute ischemic stroke in hospitalized COVID-19 patients. We evaluated risk factors for AIS in patients with COVID-19 and in a subgroup of patients with COVID-19 who developed a neurological symptom.

## Materials and Methods

### Study Design, Setting, and Population

We conducted a retrospective study of patients with COVID-19 admitted to the Yale-New Haven Health System (YNHHS) System which consists of hospitals located in the State of Connecticut, USA, including YNHH-York Street Campus (New Haven), YNHH-St Raphael's Campus (New Haven), Greenwich Hospital (Greenwich), Lawrence and Memorial Hospital (New London), and Bridgeport Hospital (Bridgeport). This study was approved by the Yale-New Haven Hospital institutional review board and informed consent was waived. Reasonable requests for access to the datasets of this study may be directed to the corresponding author.

### Identifying Patients and Outcomes

**Figure 1:**
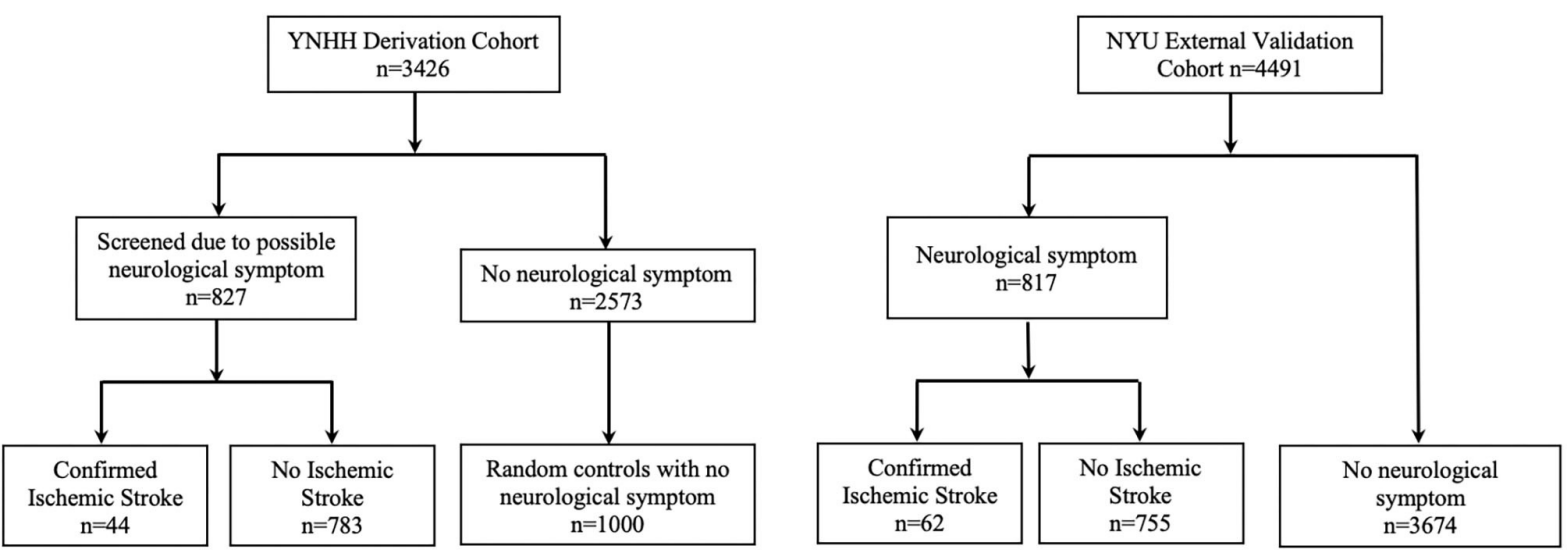
Derivation and External Validation Cohorts. YNHH, Yale New Haven Health; NYU, New York University.

A total of 3,426 patients tested positive for COVID-19 through nasopharyngeal swab by reverse-transcriptase polymerase chain reaction and were admitted to the hospital between January 3, 2020 and August 28, 2020. These hospitalized COVID-19 patients were categorized as patients (1) with an acute ischemic stroke (AIS), (2) without an AIS, and (3) without an AIS but with an acute neurological symptom. To identify AIS patients among those hospitalized with COVID-19, we first applied any of the following screening criteria to identify patients with a potential neurologic symptom: (1) a hospital ICD-10 diagnosis of ischemic stroke, intracerebral hemorrhage, subarachnoid hemorrhage, or transient ischemic attack; (2) a CT, CT angiogram, or MRI of the head performed during hospitalization; (3) a neurology consultation ordered during hospitalization, or (4) a nursing National Institutes of Health Stroke Scale assessment documented during hospitalization. A total of 827 patients met at least one of these screening criteria for neurological symptom and their medical records were individually reviewed by one of the neurologist co-authors to confirm the diagnosis of acute ischemic stroke and to determine stroke characteristics. Forty-four COVID-19 patients had confirmed AIS (42 by MRI or CT imaging and 2 through clinical examination by a board-certified neurologist). The remaining 783 patients were defined as non-AIS COVID-19 patients with an acute neurologic symptom (118 patients had neurological consultation, 93 had a brain MRI, and 581 patients had a brain CT). Among the 2,573 patients who did not meet any criteria for a potential neurologic symptom, 1,000 patients were randomly selected by simple random selection without replacement (case:control ratio > 1:5) to comprise the non-stroke control group (Figure 1) (14). Automated electronic capture was used to collect additional information on patient demographics, past medical history, clinical variables, laboratory values, imaging studies, and medications.

### Patient Characteristics

Patient features collected at the time of hospital presentation included demographics (age, gender, body mass index, and ethnicity), medical co-morbidities related to stroke (history of diabetes, hypertension, atrial fibrillation, myocardial infarction, heart valve disease, malignancy, and congestive heart failure), and initial laboratory markers of inflammation and coagulation drawn at the time of hospital presentation relevant to COVID-19 infection. These markers were selected based on prior studies and included erythrocyte sedimentation rate (ESR), C-reactive protein, D-dimer, interleukin-6 (IL-6), platelet count, procalcitonin, hemoglobin, lactate dehydrogenase, brain natriuretic peptide, and neutrophil to lymphocyte ratio (15–17). We evaluated COVID-19 severity by calculating the Quick COVID Severity Index, a validated score that uses initial respiratory rate, initial pulse oximetry value, and initial forced inspired oxygen rate to predict clinical decompensation (18).

Covariates collected during the COVID-19 hospitalization included markers of illness severity (need for mechanical ventilation, transfer to intensive care unit, requirement of extracorporeal membrane oxygenation therapy, and administration of vasopressors or heparin infusion) if these measures were utilized before the diagnosis of the AIS. Therapies for COVID-19 (corticosteroids, hydroxychloroquine, remdesivir, tocilizumab, and convalescent plasma transfusion) administered prior to the occurrence of AIS were also noted.

### External Validation Cohort

The external validation cohort consisted of patients with a diagnosis of COVID-19 admitted to one of three comprehensive stroke centers in the New York metropolitan area between March 10, 2020 and May 20, 2020. These centers are affiliated with New York University (NYU) and include NYU Langone (Manhattan), NYU Langone Brooklyn (Brooklyn), and NYU Langone Winthrop (Long Island). Similar to the derivation cohort, diagnosis of COVID-19 was determined with nasopharyngeal swab using reverse-transcriptase polymerase chain reaction. In the NYU cohort, patients with a neurology evaluation during their COVID-19 hospitalization were classified as having an acute neurological symptom. Diagnosis of AIS in the external validation cohort is identical to that of the derivation cohort, 62 COVID-19 patients had confirmed AIS (60 by MRI or CT imaging and 2 through clinical examination by a board-certified neurologist) (19). Similar to derivation cohort, demographics, clinical variables, laboratory variables, imaging variables, and in-hospital treatments were abstracted from medical records using automated electronic capture. Hospitalized COVID-19 patients were classified into those with (1) an AIS; (2) without an AIS but with an acute neurologic symptom; and (3) with neither AIS nor a neurologic symptom (Figure 1).

### Non-COVID AIS Cohort

The non-COVID AIS cohort consist of patients with AIS without COVID-19 hospitalized at Yale-New Haven Hospital consecutively from February 15, 2020 through April 28, 2020. Diagnosis of AIS was determined radiographically through MRI or CT imaging. Demographics, stroke risk factors, laboratory results, and other variables were abstracted through chart review.

### Statistical Analysis

In the derivation cohort, baseline and in-hospital characteristics of COVID-19 patients with AIS and without AIS were compared. Additionally, COVID-19 patients with AIS were compared with a subgroup of COVID-19 patients without AIS but with an acute neurologic symptom. Continuous variables were compared using non-parametric Mann-Whitney test and dichotomous variable were compared using Chi-square or Fisher exact test, as appropriate. Using covariates from univariate analyses associated with a *p*-value of < 0.20, we developed stepwise multivariable logistic regression models with entry criteria of 0.05 and stay of 0.05 classifying (1) AIS vs. non-AIS patients and (2) AIS vs. non-AIS patients with an acute neurologic symptom. Variables with more than 10% missing data were excluded from the models. Continuous variables were dichotomized at the median to fit the regression models. Certain variables were further rounded to integer values to facilitate clinical use. For each model, an area under the curve (AUC) was calculated. Significant variables were compared between COVID-19 patients with AIS and non-COVID patients with AIS.

Variables significant in the derivation cohort multivariable logistic regression models were compared between the derivation and validation cohorts by Chi- squared tests. The discriminative capacities of the derivation cohort models were tested in the respective external validation sub-cohorts.

### Sensitivity Analysis

Since the timing of AIS diagnosis may vary with respect to the time of COVID-19 hospitalization, we performed sensitivity analyses in the derivation cohort by assessing the discriminative capacity of the validated regression models in AIS cases defined (1) at the time of presentation, and (2) during the hospitalization.

Univariate analysis was performed with IBM SPSS v23 and multivariable regression and validation models were performed with SAS 9.4 (Cary, NC).

## Results

### Characteristics of the Derivation Cohort

In the YNHHS COVID-19 derivation cohort, a total of 827 patients met screening criteria for a neurologic symptom, of whom 44 patients were diagnosed with an AIS. One thousand randomly selected patients with no neurological symptoms were included in the non-stroke cohort. Patient selection details can be found in Figure 1.

Demographics, medical co-morbidities, clinical and laboratory characteristics, and markers of COVID severity for COVID-19 patients with AIS, patients without AIS, and patients without AIS but with an acute neurologic symptom are presented in Table 1. Supplementary Table 1 demonstrates additional characteristics of the AIS cohort. AIS patients had more medical co-morbidities and higher levels of markers of COVID-19 severity compared to patients without AIS. Univariate analysis comparing patients with and without AIS identified 19 variables with *p* < 0.20 (Table 1). COVID-19 AIS patients compared to COVID-19 patients without AIS were younger, had a significantly lower BMI, had higher rates of myocardial infarction, and were significantly more likely to have a prior ischemic stroke. COVID-19 AIS patients also had significantly higher admission D-dimer level, IL-6, platelet count, procalcitonin, hemoglobin, lactate dehydrogenase, brain natriuretic peptide, and neutrophil/lymphocyte ratio. AIS patients were significantly more likely to require mechanical ventilation, vasopressors, intensive care unit (ICU) admission, and to have received treatment with heparin anticoagulation prior to developing the ischemic stroke. For further analyses, we dichotomized age at 60 years, D-dimer at 1 mg/L, platelet count at 200 × 1,000/μL, and body mass index (BMI) 30.

**Table 1:** Characteristics of patients with COVID-19 with and without ischemic stroke in the derivation cohort.

**Patient characteristics**	**Overall cohort** ***N* = 1,827**	**AIS** ***N* = 44**	**Non-AIS patients** ***N* = 1,783**	**Non-AIS patients with neurological symptoms** ***N* = 783**	***P*-value^**§**^**	***P*-value^**||**^**
Average age^*^ (median, IQR)	67.0 (54.0–80.0)	64.0 (59.0–81.5)	67.0 (53.0–80.0)	61.0 (46.3–76.0)	0.303	0.200
Female gender^†^ (*n*, %)	912 (49.9%)	19 (43.2%)	893 (50.1%)	380 (48.5%)	0.366	0.490
BMI^*^ (median, IQR)	27.8 (23.8–33.2)	26.1 (22.4–28.8)	27.8 (23.8–33.3)	28.6 (24.7–34.4)	0.025	0.296
Ethnicity/race^*^ (*n*, %)					0.694	0.171
White	824 (46.5%)	19 (44.2%)	805 (46.6%)	405 (52.9%)		
Black	480 (27.1%)	12 (27.9%)	468 (27.1%)	209 (27.3%)		
Hispanic	433 (24.4%)	10 (23.3%)	423 (24.5%)	137 (17.9%)		
Asian	35 (2.0%)	2 (4.7%)	33 (1.9%)	14 (1.8%)		
Month of admission^†^					0.194	0.194
Jan–April	1,195 (65.4%)	33 (75.0%)	1,162 (65.2%)	484 (61.8%)		
May–June	538 (29.4%)	9 (20.5%)	529 (29.7%)	261 (33.3%)		
July–August	94 (5.1%)	2 (4.5%)	92 (5.2%)	38 (4.9%)		
**Medical comorbidities**						
Diabetes^†^	802 (43.9%)	23 (52.3%)	779 (43.7%)	420 (53.6%)	0.257	0.860
Hypertension^†^	590 (32.3%)	16 (36.4%)	574 (32.2%)	331 (42.3%)	0.559	0.440
Atrial fibrillation^†^	278 (15.2%)	9 (20.5%)	269 (15.1%)	165 (21.1%)	0.327	0.922
Myocardial infarction^†^	285 (15.6%)	11 (25.0%)	274 (15.4%)	159 (20.3%)	0.082	0.453
Heart valve disease^†^	326 (17.8%)	11 (25.0%)	315 (17.7%)	178 (22.7%)	0.209	0.727
Malignancy^†^	232 (12.7%)	4 (9.1%)	228 (12.8%)	113 (14.4%)	0.467	0.323
Congestive heart failure^†^	472 (25.8%)	10 (22.7%)	462 (25.9%)	273 (34.9%)	0.634	0.099
Stroke^†^	231 (12.6%)	37 (84.0%)	194 (10.9%)	194 (24.8%)	<0.001	<0.001
**Laboratory values**						
Erythrocyte sedimentation rate (mm/h)^*^	61.0 (34.5–105.0)	108.0 (43.0–111.0)	61.0 (33.8–103.3)	62.5 (39.0–106.8)	0.330	0.421
C-reactive protein (mg/L)^*^	8.4 (3.3–14.6)	10.6 (6.3–20.1)	8.3 (3.3–14.5)	7.9 (3.1–14.7)	0.207	0.218
D-Dimer (mg/L)^*^	1.1 (0.6–2.3)	2.2 (0.9–4.9)	1.1 (0.6–2.2)	1.3 (0.7–2.6)	<0.001	0.007
IL-6 (pg/mL)^*^	6.9 (5.0–19.2)	10.0 (5.0–69.0)	6.0 (5.0–19.0)	7.0 (5.0–21.0)	0.182	0.307
Platelet count (× 1,000/μL)^*^	206.0 (161.0–270.0)	256.0 (194.3– 317.0)	205.0 (160.0–269.0)	199.0 (150.0–263.0)	<0.001	0.001
Procalcitonin (ng/mL)^*^	0.13 (0.07–0.35)	0.2 (0.1–0.9)	0.13 (0.07–0.34)	0.2 (0.1–0.5)	0.069	0.340
Hemoglobin (g/dL)^*^	12.7 (11.3–14.1)	13.6 (11.7–14.6)	12.7 (11.2–14.1)	12.3 (10.9–13.8)	0.051	0.008
Lactate dehydrogenase (U/L)^*^	317.5 (239.0–425.3)	371.0 (254.0–673.0)	316.0 (238.0–422.0)	304.0 (230.3–416.3)	0.047	0.034
Brain natriuretic peptide (pg/mL)^*^	369.0 (96.8–1517.0)	938.2 (246.8–4501.5)	364.0 (94.0–1498.0)	695.0 (199.5–2629.0)	0.016	0.593
Neutrophil/lymphocyte ratio^*^	5.1 (3.1–8.7)	7.3 (4.1–13.8)	5.1 (3.1– 8.6)	5.1 (3.1–9.1)	0.001	0.003
Ejection fraction (%)^*^	61.0 (48.0–67.0)	63.0 (38.5–65.0)	61.0 (51.0–67.0)	62.0 (55.0–67.5)	0.644	0.561
QTc interval (ms)^*^	419 (395.0–441.0)	410.0 (393.0–434.0)	411.0 (390.0–435.0)	419.5 (395.1–441.5)	0.903	0.269
ABO group^†^					0.763	0.965
Blood type A	168 (32.1%)	10 (37.0%)	158 (31.8%)	86 (33.1%)		
Blood type B	76 (14.5%)	3 (11.1%)	73 (14.7%)	41 (15.8%)		
Blood type AB	13 (2.5%)	0 (0%)	13 (2.6%)	5 (1.9%)		
Blood type O	267 (51.0%)	14 (51.9%)	253 (50.9%)	128 (49.2%)		
**COVID severity**						
Mechanical ventilation^†^	308 (16.9%)	21 (45.5%)	288 (16.2%)	173 (22.1%)	<0.001	<0.001
Vasopressor use^†^	360 (19.7%)	19 (43.2%)	341 (19.1%)	206 (26.3%)	<0.001	0.014
ICU admission^†^	494 (27.0%)	24 (54.5%)	470 (26.4%)	237 (30.3%)	<0.001	0.001
Heparin anticoagulation^†^	226 (12.4%)	13 (29.5%)	213 (11.9%)	131 (16.7%)	<0.001	0.029
ECMO^‡^	8 (0.4%)	0 (0%)	8 (0.4%)	5 (0.6%)	0.656	1.000
**Quick COVID Severity Index (qCSI)**						
First respiratory rate (breaths/min)^*^	19.0 (18.0–22.0)	20.0 (18.0–23.0)	19.0 (18.0–22.0)	19.0 (18.0–22.0)	0.685	0.622
First pulse oximetry^*^	96.0 (94.0–98.0)	95.0 (92.0–98.0)	96.0 (94.0–98.0)	96.0 (94.0–98.0)	0.092	0.063
First forced inspired O_2_ rate (L/min)^*^	100.0 (96.0–100.0)	100.0 (100.0–100.0)	100.0 (96.0–100.0)	100.0 (95.0–100.0)	0.469	0.377
**COVID therapy (prior to acute ischemic stroke)**						
Corticosteroids^†^	480 (26.3%)	13 (29.5%)	467 (26.2%)	222 (28.4%)	0.618	0.864
Hydroxychloroquine^†^	1,140 (62.4%)	19 (43.2%)	1,121 (62.9%)	479 (61.2%)	0.008	0.018
Remdesivir^†^	193 (10.6%)	5 (11.4%)	188 (10.5%)	87 (11.1%)	0.861	0.959
Tocilizumab^†^	709 (38.8%)	15 (34.1%)	694 (38.9%)	305 (39.0%)	0.516	0.519
Plasma transfusion^†^	113 (6.2%)	3 (6.8%)	110 (6.2%)	59 (7.5%)	0.860	0.861
Length of Admission (days) (mean, sd)^*^	8.9 (4.6–17.3)	15.4 (10.2–23.5)	8.8 (4.5–17.1)	11.2 (5.8–22.5)	<0.001	0.030
**Outcomes**						
Hospital length of stay^*^	8.86 (4.6–17.3)	15.44 (10.2–23.5)	8.76 (4.5–17.1)	11.23 (5.8–22.5)	0.000	0.030
Death at discharge^†^	330 (18.1%)	18 (40.9%)	312 (17.5%)	189 (24.1%)	0.000	0.019

* *Mann-Whitney (median, IQR)*.

† *Chi square (n, %)*.

‡ *Fisher exact test (n, %)*.

§ *P-value comparison of ischemic stroke (n = 44) and Non-Stroke Patients (n = 1,783)*.

|| *P-value comparison of ischemic stroke (n = 44) and Non-Stroke patients with neurological symptoms (n = 783)*.

P < 0.05.

*AIS, Acute Ischemic Stroke; IQR, Interquartile Range; BMI, Body Mass Index; IL, Interleukin; ICU, Intensive Care Unit; ECMO, Extracorporeal Membrane Oxygenation*.

When comparing COVID-19 patients with AIS to COVID-19 patients without AIS but with an acute neurologic symptom, AIS patients were significantly more likely to have congestive heart failure, a prior stroke, elevated d-dimer, LDH, platelet count, hemoglobin, and neutrophil/lymphocyte ratio (Table 1). They were also more likely to require mechanical ventilation, vasopressors, ICU admission, and heparinization prior to the ischemic stroke event. With respect to COVID severity indices, the first pulse oximetry reading was higher among non-AIS COVID-19 patients with a neurologic symptom.

### Characteristics of the External Validation Cohort

The NYU external validation cohort included 4,491 patients with COVID-19, of which 817 had a neurological symptom and 62 were found to have an AIS. A total of 3,674 did not have any neurological symptoms. Patient selection details can be found in Figure 1.

### Comparison of the Two Cohorts

We compared the derivation and validation cohorts by key covariates identified by the stepwise multivariable regression models in the derivation analyses (Table 2). The COVID-19 ischemic stroke patients of the derivation and external validation cohort had similar baseline characteristics, laboratory values at presentation, and hospital course with exception of BMI (*p* = 0.037), hemoglobin level (*p* < 0.001), and D-dimer level (*p* < 0.001).

**Table 2:** Characteristics of COVID-19 ischemic stroke patients in the derivation and external validation cohorts.

**Characteristics**	**Derivation cohort ischemic stroke patients** ***N* = 44 (*n*, %)**	**External validation ischemic stroke patients** ***N* = 62 (*n*, %)**	***P*-value**
**Baseline characteristics**			
Age <60 years	15/44 (34.1%)	20/62 (32%)	*P* = 0.843
Female sex	19/44 (43.2%)	18/62 (29%)	*P* = 0.132
BMI > 30	9/43 (20.9%)	25/62 (40%)	*P* = 0.037
History of prior stroke	37/44 (84.1%)	44/62 (71%)	*P* = 0.117
History of myocardial infarction	11/44 (25%)	10/61 (16%)	*P* = 0.277
**Laboratory values at presentation**			
Hemoglobin (g/dL) > 12	30/44 (68.2%)	11/43 (18%)	*P* < 0.001
Platelet count (× 1,000/μL) > 200	33/44 (75.0%)	26/43 (42%)	*P* = 0.147
D-Dimer (mg/L) > 1	31/42 (73.8%)	17/51 (27%);	*P* < 0.001
**Hospital course**			
Admitted to ICU^*^	24/44 (54.5%)	42/62 (68%)	*P* = 0.167
Mechanical ventilation^*^	20/44 (45.5%)	40/62 (65%)	*P* = 0.051
Hydroxychloroquine use^*^	33/44 (75.0%)	52/62 (84%)	*P* = 0.259

* *Prior to ischemic stroke*.

*BMI, Body Mass Index; ICU, Intensive care unit*.

#### Comparing COVID-19 Patients With and Without AIS

In the derivation cohort, history of stroke (OR 41.43, 95% C.I. 18.05–95.08; *p* < 0.001) and platelet count at presentation > 200 × 1,000/μL (OR 2.85, 95% C.I. 1.37–5.93, *p* = 0.005) were independent predictors of ischemic stroke during COVID-19 hospitalization (Table 3). There were no other significant baseline characteristics or in-hospital predictors including clinical indicators of COVID-19 severity and therapies implemented during hospitalization (Supplementary Table 2). In the external validation cohort, only having a prior history of stroke achieved statistical significance (*p* < 0.001). The derivation AUC of this model was 0.89 and the external validation AUC of this model was 0.82. In the external validation model, history of stroke alone achieved an AUC of 0.84. In the sensitivity analysis of patients diagnosed with AIS at the time of presentation, history of stroke, and platelet count > 200 × 1,000/μL maintained significance (AUC 0.88). In the sensitivity analyses, history of stroke and platelet count > 200 × 1,000/μL predicted having an AIS vs. not both among patients diagnosed with AIS at the time of presentation and those diagnosed with AIS during the hospitalization (AUC 0.88 and 0.88, respectively, Supplementary Table 3).

**Table 3:** Predictors of acute ischemic stroke vs. no ischemic stroke in the derivation and validation cohorts.

	**Derivation cohort** **(AIS** ***N*** **=** **44, total** ***N*** **=** **1,827)**			**External validation cohort** **(AIS** ***N*** **=** **62, total** ***N*** **=** **4,491)**		
**Variable**	**OR (95% CI)**	***P*-value**	**AUC**	**OR (95% CI)**	***P*-value**	**AUC**
History of prior stroke	41.43 (18.05–95.08)	<0.001	0.89	40.12 (19.34–82.30)	<0.001	0.82^*^
Platelet count at presentation ≥ 200 × 1,000/μL	2.85 (1.37–5.93)	0.005		1.18 (0.60–2.30)	0.635	

* *The AUC for history of stroke alone was 0.84*.

*AIS, Acute Ischemic Stroke; OR, Odds ratio; CI, Confidence Interval; AUC, Area under curve*.

#### Comparing COVID-19 Patients With AIS and Patients Without AIS but With a Neurologic Symptom

In the derivation cohort, history of stroke (OR 35.78, 95% C.I. 7.23–176.95; *p* < 0.001) and age ≤ 60 (OR 3.75, 95% C.I. 1.36–10.31; *p* < 0.012) were independent predictors of AIS during hospitalization among COVID-19 patients with an acute neurologic symptom (Table 4). This model was externally validated with similar statistical significance for history of prior stroke (OR 19.2, 95% C.I. 10.40–35.50; *p* < 0.001) and age ≤ 60 years (OR 2.31, 95% C.I. 1.20–4.45; *p* = 0.012). The derivation AUC was 0.83 and the external validation AUC was 0.81. In the sensitivity analyses, history of stroke and age ≤ 60 years predicted having an AIS vs. not both among patients with a neurologic symptom diagnosed with AIS at the time of presentation and those diagnosed with AIS during the hospitalization (AUC 0.80 and 0.79, respectively, Supplementary Table 4).

**Table 4:** Predictors of acute ischemic stroke vs. no ischemic stroke among patients with a neurologic symptom in the derivation and validation cohorts.

	**Derivation cohort** **(AIS** ***N*** **=** **44, total** ***N*** **=** **827)**			**External validation cohort** **(AIS** ***N*** **=** **62, total** ***N*** **=** **817)**		
**Variable**	**OR (95% CI)**	***P*-value**	**AUC**	**OR (95% CI)**	***P*-value**	**AUC**
History of prior stroke	35.78 (7.23–176.95)	<0.001	0.83	19.2 (10.40–35.50)	<0.001	0.81
Age ≤ 60	3.75 (1.36–10.31)	0.011		2.31 (1.20–4.45)	0.012	

*AIS, Acute Ischemic Stroke; OR, Odds ratio; CI, Confidence Interval; AUC, Area under curve*.

#### Comparing COVID-19 Patients With AIS and Non-COVID Patients With AIS

To evaluate whether predictors of AIS were specific for patients with COVID-19, we compared these predictors between AIS patients with and without COVID-19. Characteristics of non-COVID patients with AIS can be seen in Supplementary Table 5. Our analysis shows that COVID-19 patients with acute ischemic stroke are younger (*p* < 0.0001) and more likely to have a prior stroke (*p* = 0.001), but not more likely to have increased or decreased platelets (*p* = 0.372) (Table 5).

**Table 5:** Comparing COVID-19 ischemic stroke vs. non-COVID 19 ischemic stroke patients.

**Patient characteristic**	**COVID-19 ischemic stroke patients** ***N* = 44** **(*n*, %)**	**Non-COVID-19 ischemic stroke patients** ***N* = 168** **(*n*, %)**	***P*-value**
Age <60 years	28 (63.6%)	40 (23.8%)	<0.0001
History of prior stroke	37 (84.1%)	37 (22.2%)	<0.0001
Platelet count > 200 × 1,000/μL	33 (75.0%)	104 (68.0%)	0.3721

## Discussion

Using derivation and validation cohorts, we identified predictors of acute ischemic stroke in hospitalized COVID-19 patients. Among all COVID-19 patients, history of a prior stroke alone was predictive of an AIS during COVID-19 hospitalization in both cohorts. In patients with a neurologic symptom, a prior history of stroke and younger age were independent risk factors for AIS. These findings may have clinical implications for evaluating the risk of ischemic stroke in COVID-19 patients with or without a new neurologic symptom that may guide further diagnostic workup and management.

The characteristics of our derivation stroke cohort are similar to those described in the literature. Patients with AIS had a median age of 64 and higher rates of co-morbidities such as hypertension, diabetes, and coronary artery disease (20, 21). Similar to other studies, our ischemic stroke cohort, when compared to non-stroke patients, also had higher markers of inflammation and coagulation such as elevated IL-6, procalcitonin, erythrocyte sedimentation rate, and D-dimer, as well as more signs of COVID-19 illness severity such as ICU admission or mechanical ventilation (22, 23). However, these markers of inflammation and COVID-19 severity were not predictive of AIS in any of our models. The mechanism by which COVID-19 causes ischemic stroke and other neurological manifestations may be related to hypercoagulability and endothelial injury but it is likely multifactorial (3, 24, 25).

Among all COVID-19 patients hospitalized in the derivation cohort, prior history of stroke and platelets >200 × 1,000/μL were the only covariates predictive of AIS. The association between prior stroke and risk of future stroke has been well- established and may continue to play a role in predicting stroke in patients with COVID-19 (26, 27). Platelets have also been implicated in COVID-19 pathophysiology, becoming hyperactive during infection (28–30). Elevation of platelets on admission has been shown to be predictive of thrombosis during hospitalization, and more recent studies suggest that the use of aspirin may decrease in-hospital mortality (29, 31, 32). Our study found that admission platelets of >200 × 1,000/μL were predictive of AIS, but this covariate was not validated through the NYU cohort. The lack of predictive value of platelets in ischemic stroke in this study might be due to institutional differences regarding the use of thromboprophylaxis between the YNHHS and NYU cohorts, both of which have undergone several modifications throughout the COVID-19 pandemic (3, 33–35).

In a cohort of patients with COVID-19 and neurological symptoms, history of prior stroke and age <60 years old were independent predictors of AIS. Both of these covariates were externally validated using the NYU population. The association between younger age and stroke has been previously described in COVID-19 populations. The average age of non-COVID-19 patients with ischemic stroke is around 74 years old; however, multiple cohorts worldwide have found that the average age of patients with COVID-19 and concurrent ischemic stroke is substantially younger, ranging between 54.9 and 64 years old (7, 36–39). It is unclear why COVID-19 increases stroke risk in younger individuals out of proportion to what would be expected in non-COVID-19 cohorts, but we hypothesize that younger patients may be more susceptible to endothelial injury as we note in our previously published study from either the direct infection or an post-infectious immunologic pathway that needs to be further elucidated (5). There may also be selection bias at play, as older patients with COVID-19 may be more likely to withdraw care and less likely to undergo further stroke testing. Patients with COVID-19, neurological symptoms, a prior stroke, and younger age should have a lower threshold to undergo evaluation for suspected AIS.

Our predictive models have several clinical implications. The identification of covariates predictive of ischemic stroke may enable clinicians to determine which patients are at higher risk of stroke and ensure adequate triaging and diagnostic workup. In particular, our models suggest that patients with COVID-19, concurrent neurological symptoms, a prior history of stroke, and age of less than or equal to 60 years old should have a lower threshold to undergo evaluation for suspected AIS. Additional factors such as elevated platelets and other markers of COVID-19 illness severity may further increase the suspicion for ischemic stroke. Further studies are needed to further validate our models and to determine other variables predictive of ischemic stroke in patients with COVID-19.

Our study has several limitations. Due to institutional restrictions pertaining to data on patients with COVID-19, our derivation cohort could only include 1,000 random controls rather than the entire population of 2,573 patients with no neurological symptoms, which may have underpowered our study. Second, due to the severity of COVID-19 and the shortage of diagnostic resources during the early stages of this global pandemic, we suspect that our derivation cohort may be underestimating the true rate of ischemic stroke. Early transition to comfort measures only, restricted access to magnetic resonance neuro-imaging studies, and limited neurological evaluation due to restricted patient access may have adversely impacted the opportunities to diagnose all cases of ischemic stroke. This is a retrospective, observational study in which clinical and laboratory studies relevant to stroke might have not been obtained for every patient, resulting in missingness. Furthermore, there were differences in the methods used to determine the occurrence of a neurologic symptom in the YNHHS and the NYU cohorts. Due to differences in COVID-19 treatment and disease monitoring algorithms used by hospital systems across the world, it is possible that the findings in our study may not be generalizable to other hospitals. This model does not predict AIS in all COVID patients and is limited to patients hospitalized with COVID-19. It is also important to note that the cohort in this study was from early in the pandemic and its generalizable to the current times is unknown.

## Conclusion

We identified several independent risk factors of acute ischemic stroke in COVID-19 hospitalized patients. COVID-19 patients with an AIS were more likely to have a prior ischemic stroke and elevated platelet counts at presentation, regardless of clinical COVID-19 disease severity and treatment metrics. Among COVID-19 patients with a neurological symptom, history of prior ischemic stroke and age <60 alone were predictive of AIS. These covariates are readily available and may facilitate the diagnosis of acute ischemic stroke in patients with COVID-19. However, additional studies are necessary to confirm the findings of this study.

## Data Availability Statement

The raw data supporting the conclusion of this article can be made available by authors at request, without undue reservation.

## Ethics Statement

The studies involving human participants were reviewed and approved by Yale University IRB, New York University IRB. Written informed consent for participation was not required for this study in accordance with the national legislation and the institutional requirements.

## Author Contributions

All authors listed have made a substantial, direct and intellectual contribution to the work, and approved it for publication.

## Funding

JF and SY report grant support from NIH/NIA grant 3P30AG066512-01S1 and NIH/NINDS grant 3U24NS113844-01S1. KS was supported by the NIH (U24NS107136, U24NS107215, R01NR018335, R01NS107215, U01NS106513, and R03NS112859) and the AHA (18TPA34170180 and 17CSA33550004).

## Conflict of Interest

SS directs a study within the AIDS Clinical Trials Group that receives study medications donated by ViiV Healthcare, Inc. KS reports research grant funding from Biogen, Novartis, Bard, Hyperfine, is on the data and safety monitoring board for a study from Zoll, and reports equity in Alva Health. The remaining authors declare that the research was conducted in the absence of any commercial or financial relationships that could be construed as a potential conflict of interest.

## Publisher's Note

All claims expressed in this article are solely those of the authors and do not necessarily represent those of their affiliated organizations, or those of the publisher, the editors and the reviewers. Any product that may be evaluated in this article, or claim that may be made by its manufacturer, is not guaranteed or endorsed by the publisher.
