# Supplementary material for: Prior Stroke and Age Predict Acute Ischemic Stroke Among Hospitalized COVID-19 Patients: A Derivation and Validation Study
Source: Front Neurol. 2021 Oct 4;12:741044. doi: 10.3389/fneur.2021.741044 (PMC8524436; doi:10.3389/fneur.2021.741044)
Supplement: Supplementary file 1 [file Data_Sheet_1.docx]

**Supplementary Table 1: Characteristics of the Derivation Acute Ischemic Stroke Cohort (N=44)**

|  | COVID patients with AIS (n=44) |
| --- | --- |
| Average Age (IQR) | 64.0 (59.0 - 81.5) |
| Female gender (n, %) | 19 (43.2%) |
| Home antiplatelet (n, %) | 21 (47.7%) |
| Home anticoagulation (n, %) | 9 (20.5%) |
| Home statin (n, %) | 25 (56.8%) |
| mRS pre admission (n, %) |  |
| 0 | 18 (40.9%) |
| 1 | 5 (11.4%) |
| 2 | 1 (2.3%) |
| 3 | 2 (4.5%) |
| 4 | 6 (13.6%) |
| 5 | 2 (4.5%) |
| Initial NIHSS |  |
| 0-4 | 7 (30.3%) |
| 5-9 | 5 (21.6%) |
| 10-14 | 4 (17.3%) |
| 15-20 | 7 (30.3%) |
| Stroke Location (n, %) |  |
| Thalamus | 3 (6.8%) |
| Basal ganglia | 4 (9.1%) |
| Cortical | 30 (68.2%) |
| Brainstem | 1 (2.3%) |
| Cerebellum | 1 (2.3%) |
| Hemorrhagic conversion (n, %) | 6 (13.6%) |
| TOAST criteria |  |
| Large artery atherosclerosis | 3 (6.8%) |
| Cardioembolism | 8 (18.2%) |
| Small vessel | 1 (2.3%) |
| Other cause | 5 (11.4%) |
| Stroke of undetermined etiology | 26 (59.1%) |
| Large Vessel Occlusion (n, %) | 9 (20.5%) https://9gag.com/gag/aV71j0d |
| Received tPA (n, %) | 1 (2.3%) |
| Endovascular therapy pursued (n, %) | 1 (2.3%) |
| mRS at discharge (n, %) |  |
| 0 | 3 (6.8%) |
| 1 | 3 (6.8%) |
| 2 | 2 (4.5%) |
| 3 | 2 (4.5%) |
| 4 | 6 (13.6%) |
| 5 | 8 (18.2%) |
| 6 | 16 (36.4%) |

*AIS=Acute Ischemic Stroke, IQR= Interquartile Range, mRS= modified Ranking Scale, NIHSS= National Institutes of Health Stroke Scale, TOAST= Trial of Org 10172 in Acute Stroke Treatment, tPA= Tissue plasminogen activator

**Supplementary Table 2:**

Predictors of ischemic stroke among COVID-19 patients with and without AIS and among COVID-19 patients with a neurological symptom.

| All COVID-19 patients with and without AIS | | |
| --- | --- | --- |
| Variable | OR (95% CI) | P-Value |
| Age | 4.08 (1.29 – 12.87) | 0.001 |
| D-dimer | 2.02 (0.72 – 5.62) | 0.181 |
| Prior heart failure | 3.37 (0.81 – 13.94) | 0.094 |
| Lactate dehydrogenase | 0.56 (0.20 – 1.58) | 0.274 |
| Neutrophil/Lymphocyte ratio | 0.80 (0.29 – 2.22) | 0.669 |
| Platelet count | 0.44 (0.16 – 1.23) | 0.119 |
| Race- White | 1.15 (0.36 – 3.66) | 0.684 |
| Race- Black | 0.86 (0.22 – 3.43) | 0.389 |
| Race- Hispanic | 4.22 (0.16 – 108.4) | 0.379 |
| History of stroke | 20.52 (6.61 – 63.69) | <0.001 |
| COVID-19 patients with a neurological symptom | | |
| Variable | OR (95% CI) | P-Value |
| Body mass index | 1.39 (0.62 – 3.12) | 0.424 |
| D-dimer | 0.63 (0.30 – 1.32) | 0.226 |
| Myocardial infarction | 1.16 (0.54 – 2.52) | 0.702 |
| Neutrophil/Lymphocyte ratio | 0.66 (0.32 – 1.37) | 0.264 |
| Platelet count | 2.69 (1.27 – 5.71) | 0.009 |
| Procalcitonin | 0.86 (0.41 – 1.78) | 0.676 |
| History of stroke | 39.61 (16.91 – 92.78) | <0.001 |

**Supplementary Table 3:**

Predictors of acute ischemic stroke at hospital presentation (N=20) versus no ischemic stroke and acute ischemic stroke during hospitalization (N=24) versus no ischemic stroke in the derivation cohort.

|  | **Patients presenting with AIS**  **(N= 20, total N=1827)** | | | **Patients with AIS during hospitalization**  **(N=24, total N=1827)** | | |
| --- | --- | --- | --- | --- | --- | --- |
| **Variable** | **OR (95%CI)** | **P value** | **AUC** | **OR (95%CI)** | **P value** | **AUC** |
| History of prior stroke | 33.13  (12.27–89.46) | <0.001 | 0.88 | 31.51  (11.60–85.57) | <0.001 | 0.88 |
| Platelet count at presentation ≥ 200 x1000 /µL | 2.38  (0.96–5.86) | 0.060 |  | 2.77  (1.07–7.16) | 0.036 |  |

*AIS= Acute Ischemic Stroke, OR= Odds ratio, AUC= Area under curve

**Supplementary Table 4:**

Predictors of acute ischemic stroke at hospital presentation (N=20) among patients with a neurological symptom and acute ischemic stroke during hospitalization (N=24) among patients with neurological symptoms in the derivation cohort.

|  | **Patients presenting with AIS**  **(N= 20, total N=1827)** | | | **Patients with AIS during hospitalization**  **(N=24, total N=1827)** | | |
| --- | --- | --- | --- | --- | --- | --- |
| **Variable** | **OR (95%CI)** | **P value** | **AUC** | **OR (95%CI)** | **P value** | **AUC** |
| History of prior stroke | 13.25  (4.86–36.10) | <0.001 | 0.80 | 12.66  (4.62–34.70) | <0.001 | 0.79 |
| Age ≤ 60 | 2.46  (1.03–5.88) | 0.042 |  | 2.61  (1.09–6.29) | 0.032 |  |

*AIS= Acute Ischemic Stroke, OR= Odds ratio, AUC= Area under curve

**Supplementary Table 5: Characteristics of the Derivation Acute Ischemic Stroke Cohort Without COVID-19 (N=168)**

|  | Non-COVID patients with AIS (n=168) |
| --- | --- |
| Average Age (median, IQR) | 71 (61– 84) |
| Female gender (n, %) | 74 (44.1%) |
| Race |  |
| White (n, %) | 115 (73.3%) |
| Black (n, %) | 35 (22.3%) |
| Other (n, %) | 7 (4.5%) |
| Ethnicity |  |
| Hispanic (n, %) | 10 (6.2%) |
| Non-Hispanic (n, %) | 152 (93.8%) |
| Ischemic stroke risk factors |  |
| Hypertension (n, %) | 127 (75.6%) |
| Hyperlipidemia (n, %) | 114 (67.9%) |
| History of smoking (n, %) | 84 (50.6%) |
| Diabetes (n, %) | 63 (37.5%) |
| Atrial fibrillation (n, %) | 52 (31.1%) |
| History of stroke (n, %) | 37 (22.2%) |
| Antithrombotic medications at onset |  |
| Antiplatelet (n, %) | 65 (38.7%) |
| Anticoagulation (n, %) | 32 (19.1%) |
| NIHSS at presentation (median, IQR) | 6 (2–14) |
| Large vessel occlusion (n, %) | 77 (45.8%) |
| Suspected stroke etiology |  |
| Large artery atherosclerosis (n, %) | 25 (15.7%) |
| Cardioembolism (n, %) | 50 (31.5%) |
| Small Vessel occlusion (n, %) | 20 (12.6%) |
| Other determined etiology (n, %) | 15 (9.4%) |
| Undetermined etiology (n, %) | 49 (30.8%) |
| Emergent stroke treatment |  |
| Thrombectomy (n, %) | 25 (15.9%) |
| tPA (n, %) | 52 (31.0%) |

*AIS=Acute Ischemic Stroke, IQR= Interquartile Range, NIHSS= National Institutes of Health Stroke Scale, tPA= Tissue plasminogen activator
